# Bile Salt Hydrolase Activity in the Food-Derived Strain *Levilactobacillus brevis* M3R3: Genomic and Functional Characterization

**DOI:** 10.1007/s12602-025-10810-0

**Published:** 2025-11-19

**Authors:** Gianluigi Agolino, Marianna Cristofolini, Maria Anna Ronsivalle, Alice Cattivelli, Davide Tagliazucchi, Cinzia Caggia, Lisa Solieri, Cinzia L. Randazzo

**Affiliations:** 1 Department of Agricultural, Food and Environment, University of Catania, 100, 95123 Catania, Italy; 2 Department of Life Sciences, University of Modena and Reggio Emilia, Besta Building, Via Amendola 2, 42122 Reggio Emilia, Italy; 3 Probioetna SRL, Spin off of, University of Catania , Via S. Sofia 100, 95123 Catania, Italy

**Keywords:** Bile salt hydrolase, Whole genome, Glyco-conjugated BSs, *bsh* genes

## Abstract

In the present study, *Levilactobacillus brevis* M3R3, isolated from traditional Sicilian sourdough, was evaluated for bile salt hydrolase (BSH) activity through kinetic growth assays and bile salts (BSs) deconjugation analysis by UHPLC/HR-MS. Genome sequencing and in silico analyses were performed to assess its safety and probiotic potential. Results showed that strain M3R3 tolerated different concentrations of individual conjugated BSs, with dose-dependent growth inhibition. Exposure to 1.0% glycocholic and glycodeoxycholic acids reduced M3R3 growth, indicating an adaptive stress response. When exposed to a mixed BS solution mimicking intestinal conditions, the strain maintained growth despite an extended lag phase and a reduced final biomass, likely due to toxic BSs accumulation. UHPLC/HR-MS confirmed complete deconjugation of glyco-conjugated BSs and partial deconjugation of tauro-conjugated BSs. Whole-genome sequencing (2.30 Mbp, 2375 CDSs) revealed absence of antimicrobial resistance or virulence genes and identified two *bsh* genes (*bsh_2A* and *bsh_3A*), both expressed under control and BS-exposed conditions. Phylogenetic analysis of *L. brevis* BSH proteins identified three clusters (I, II, and III), with *bsh_2A* and *bsh_3A* from M3R3 grouped in clusters II and III, respectively. Overall, genomic and functional characterizations support that *L. brevis* M3R3 is a safe, BSH-active strain, with potential applications as a functional ingredient in health promoting formulations.

**Supplementary Information:**

The online version contains supplementary material available at 10.1007/s12602-025-10810-0.

## Introduction

Lactobacilli strains of food origin are widely recognized for their capacity to support gastrointestinal health by modulating host functions and contributing to the maintenance of intestinal microbiota homeostasis. To exert these beneficial effects, they must withstand the harsh conditions of the gastrointestinal tract, including gastric acidity and bile salts [1].

Bile salts (BSs) are endogenous signaling molecules synthesized from cholesterol by hepatocytes. In the liver, cholesterol undergoes a multistep metabolic pathway to form primary bile acids, which are subsequently conjugated with glycine or taurine to increase their solubility [2]. Upon secretion into bile, these conjugated acids are associated with cations such as Na^+^ and K^+^, forming BSs [3]. Primary BSs represent the initial product of cholesterol catabolism. Their conjugation with glycine and taurine occurs through the formation of an amide bond between the bile acid’s carboxyl group and the amino group of the respective amino acid, a modification essential for efficient lipid emulsification and other physiological functions [3]. Once released into the small intestine, part of primary BSs can be deconjugated by resident bacterial communities and further converted into secondary BSs, a process that also influences the gut microbiota composition.

Bile salt hydrolase (BSH) (choloylglycine hydrolase; EC 3.5.1.24) is the microbial enzyme which plays a crucial role in BSs metabolism by cleaving the amide bond of conjugated BSs, thereby releasing unconjugated bile acids with free taurine or glycine [4]. BSH activity is widely distributed among gastrointestinal microbes, including various *Lactobacillaceae* species [5–8], where the presence of multiple *bsh* orthologs reflects strain-specific adaptations into the intestinal environment [5, 8–10]. BSH-positive bacteria provide an important contribution to host bile detoxification [6]. The deconjugation ability enhances bacterial survival and colonization of the gastrointestinal tract, modulates lipid metabolism, and shapes the bile acid pool, ultimately influencing host-microbiome signaling [6, 10, 11]. Among its metabolic benefits, BSH activity has been implicated in the regulation of host cholesterol levels, contributing to its potential cholesterol-lowering effect [11–13]. Moreover, BSH is associated with other beneficial functions including the regulation of energy balance and glucose metabolism [14].

Considering these physiological roles, increasing attention has been directed toward isolating novel BSH-active strains. Fermented foods are rich sources of probiotic candidates, including BSH-active lactobacilli. Although BSH activity is predominantly associated with gastrointestinal isolates, non-intestinal lactobacilli from fermented foods also show deconjugation ability and thus potential probiotic value [15–17]. Among these, *Levilactobacillus brevis* has attracted attention due to its functional and probiotic properties [18–23]. It has been granted Qualified Presumption of Safety (QPS) status, underscoring its safety for food applications [24]. Technologically, *L. brevis* contributes to bioactive compound production, enhancement of aroma and flavor, and improved fermentation outcomes [25–27]. Genomic analyses indicate that *L. brevis* carries multiple *bsh* gene copies of different subtypes [9], consistent with observed bile tolerance and cholesterol‐lowering activity in vitro [28–30].

The present study aimed to investigate a *L. brevis* strain isolated from traditional Sicilian sourdough, with the objective of proposing it as a novel BSs-modulating probiotic. To elucidate the mechanisms underlying the BSH activity, phenotypic assays were employed to verify BSs tolerance and deconjugation ability. A combination of genomic and phylogenetic analyses was also performed to characterize the presence and distribution of BSH-related genes inside the *L. brevis* M3R3 strain.

## Materials and Methods

### Bacterial Strains and Culture Conditions

*Levilactibacillus brevis* M3R3, previously isolated from sourdough, is part of the microbial culture collection of ProBioEtna srl, a spin-off of the University of Catania, Italy. The strain was routinely propagated anaerobically in de Man, Rogosa and Sharpe (MRS) medium (Oxoid, Basingstoke, Hampshire, UK) at 37 °C for 48 h in a 2.5-L anaerobic jar (Cat. No. AG0025A; Thermo Fisher Scientific, Waltham, MA, USA). For long-term preservation, the strain was cryopreserved at − 80 °C using the same cultivation medium supplemented with 25% (*v/v*) glycerol.

### Bile Salt Tolerance Assay

Bile salt tolerance of *L. brevis* strain M3R3 was assessed under static conditions in MRS broth supplemented separately with taurodeoxycholic acid (TDCA; Cat No. 580221), taurocholic acid (TCA; Cat. No. 580217), glycodeoxycholic acid (GDCA; Cat. No. 361311), and glycocholic acid (GCA; Cat. No. 360512) sodium salts at the final concentrations of 0.1% 0.25%, 0.5%, and 1.0% (*w/v*), respectively. All BSs were purchased from Sigma Aldrich (St. Louis, MO, United States). MRS medium without BSs supplementation served as the control. Specifically, exponentially growing cells of strain M3R3 were inoculated into 5 mL of each modified MRS medium at the final density of 1.0 × 10^5^ CFU/mL and incubated anaerobically at 37 °C for 24 h. Bacterial counts were determined by plating serial ten-fold dilutions of each bacterial culture onto MRS agar. After the incubation of plates at 37 °C for 48 h under anaerobic conditions, colonies were enumerated from plates containing 20–200 colonies. The results were expressed as Log_10_ CFU/mL in the presence of each BS relative to the control.

### Growth Curve Assays

Growth kinetics were assessed under static conditions according to Agolino and co-worker [31]. Specifically, *L. brevis* M3R3 was inoculated in triplicate into MRS broth supplemented with 1.0% (*w/v*) of each BS (TDCA, TCA, GDCA, and GCA) separately or a BSs mixture (Oxoid, Basingstoke, Hampshire, UK) at the final density of 1.0 × 10^5^ CFU/mL. MRS medium without supplementation was used as the control. Microbial growth was monitored by measuring optical density at 600 nm (OD_600nm_) in three technical replicates recorded at least three times per day.

Kinetic parameters were determined by fitting the experimental data to either parametric or non-parametric smooth spline models provided by the Grofit package (version 1.1.1–1) in R [32]. The best fit model was selected based on the Akaike Information Criterion (AIC). For each curve, the following parameters were derived: maximum growth rate (µ, expressed as h⁻^1^), lag phase duration (λ, indicated as h), and maximum cell density reached at stationary phase (A, OD_600nm_). During the late stationary phase of each growth curve assay, cultures were centrifuged at 8000× *g* for 10 min under refrigerated conditions to obtain and cell-free supernatants for subsequent UHPLC/HR-MS analysis. In the case of growth assays in presence of BSs mixture, cells pellets were collected for RNA extraction. All the samples were stored at −80 °C until further use.

### UHPLC/HR-MS Analysis

Ultra-high-performance liquid chromatography high-resolution mass spectrometry (UHPLC/HR-MS) analysis was performed, according to the method described by Karlov and co-worker [33], with slight modifications. Chromatographic separation was performed in UHPLC Ultimate 3000 system (Thermo Fisher Scientific, San Jose, CA, USA), while compound identification and semi-quantitative analysis were carried out with a Q Exactive Hybrid Quadrupole-Orbitrap Mass Spectrometer (Thermo Fisher Scientific, San Jose, CA, USA). Prior to analysis, samples were appropriately diluted, and 10 μL were injected into the UHPLC system loaded using a a C18 reversed-phase column Acquity UPLC HSS C18, 2.1 × 100 mm, 1.8 μm particle size (Waters, Milan, Italy). The mobile phases consisted of water with 0.1% formic acid (solvent A) and acetonitrile with 0.1% formic acid (solvent B). The gradient elution started at 58% B, increased linearly to 75% over 10 min, followed by a rapid increase to 98% B within 1 min. This condition was maintained for 6 min before re-equilibrating to the initial conditions. The flow rate was maintained at 0.3 ml/min and the column temperature was set at 45 °C. Mass spectrometry analysis was carried out under negative electrospray ionization (ESI) mode using the following parameters: capillary voltage 2.7 kV; capillary temperature, 320 °C; sheath gas flow rate, 40 (arbitrary units); and auxiliary gas flow rate, 30 (arbitrary units). Full MS scans were acquired at a resolution of 70,000 with an AGC target of 3 × 10⁶, a maximum injection time (IT) of 247 ms, and a scan range of 100–1500 m/z. MS/MS scans were performed at a resolution of 16,500, with an AGC target of 5 × 10^5^, a maximum IT of 120 ms, and an isolation window of 1 m/z.

Identification of the different BSs was carried out by comparing the retention time, the m/z value and the fragmentation pattern with authentic standard compounds, as previously reported [31]. The relative amount of the residual compound was quantified by integrating the area under the corresponding chromatographic peaks (AUC) and calculated using the equation as follows:

$$\%\;\mathrm{decrease}\;=\;100-\left[100\times\left(\mathrm{AUP}\;\mathrm{of}\;\mathrm{BA}\;\mathrm{in}\;\mathrm{inoculated}\;\mathrm{sample}/\mathrm{AUP}\;\mathrm{of}\;\mathrm{BA}\;\mathrm{in}\;\mathrm{standard}\;\mathrm{solution}\right)\right]$$

### Genome Sequencing

The genomic DNA extraction was carried out as previously reported [31]. Library preparation and genome sequencing were outsourced to BMR Genomics (Padua, Italy). Genomic DNA was sequenced using the Illumina MiSeq platform with the V3 reagent kit, generating 2 × 300 bp paired-end reads. Pre-processing step was carried out with Fastq v0.23.2 [34] to remove adapters and filter low quality bases (Q < 30) and short reads (length < 150). Possible contaminants were assessed by MetaPhlan v4.0.1 [35]. SPAdes v3.15.5 [36] was used to perform de novo genome assembly on the cleaned reads. QUAST v5.2.0 [37] and BUSCO v5.4.3 [38] were used to evaluate genome completeness and assembly quality.

### Gene Prediction and Functional Annotation

Gene prediction and annotation were carried out with Prokka v1.14.6 [39]. Graphical representation of the M3R3 genome was generated using the Proksee Server (v1.1.2) based on GenBank-formatted annotation files [40]. The cluster of orthologous groups (COG) for the protein-coding genes was obtained using Egg-NOG mapper (v2.1.7) tool [41] from the online Egg-NOG database (v5.0). A complementary functional analysis was performed using the Kyoto Encyclopedia of Genes and Genomes (KEGG) mapper/BLASTKOALA tool [42].

### Taxonomic Identification and Comparative Genomics

16S rRNA gene sequencing and phylogenetic analysis on was carried out as previously reported [43]. The 16S rRNA gene sequence of strain M3R3 was deposited in GenBank under the accession number PV902533. Species identification of strain M3R3 was determined via calculation of the Average Nucleotide Identity (ANI) index using the JSpecies web server tool with default parameters [44] and calculation of the digital DNA–DNA hybridization (dDDH) values using an online Genome-to-Genome Distance Calculator (GGDC3.0; https://ggdc.dsmz.de/ggdc.php) with formula 2 [45]. The species thresholds of 95% and 70% were considered for ANI and dDDH analyses, respectively [46]. In addition, the Genome BLAST Distance Phylogeny (GBDP) was constructed in the Type Strain Genome Server (TYGS) to infer the taxonomical affiliation at the species level for strain M3R3 [45].

For species-specific phylogeny, a total of 178 *L. brevis* genomes were retrieved from the NCBI database (last accessed January 2024). Four genomes exhibited a completeness lower than 85% and/or a contamination level above 3%, as assessed by CheckM, and were excluded from the analysis, resulting in a final dataset of 174 high-quality genomes (Table S1). Core genome single nucleotide polymorphisms (SNPs) were identified using Snippy-multi v4.6.0 (https://github.com/tseemann/snippy). Reads and assembled genomes were mapped against the selected *L. brevis* reference genome from strain NCTC13768 (GCA_900475625.1). Recombination regions in the core genome alignment were detected and masked using Gubbins v2.4.1 to reduce phylogenetic bias [47]. RAxML program was used to build the phylogenetic tree using the clean core SNP alignments generated from Gubbins.

A subset of 10 *L. brevis* genomes was included in the subsequent core-genome and pan-genome analyses. In detail, core-genome and pan-genome were computed in the Efficient Database framework for comparative Genome Analyses using BLAST score Ratios (EDGAR v3.2) web tool, which checked for reciprocal best BLAST hits against all other genomes with *L. brevis* strain M3R3 serving as the reference genome [48]. Since EDGAR uses a bidirectional best BLASTp hits approach for calculating singletons, gene duplication and paralogs are treated as singletons, if unidentical [49]. All trees generated in this study were visualized using the Interactive Tree Of Life (iTOL v7) [50].

### In Silico Safety and Functional Assessments

In silico safety assessments were conducted in line with recommendations from the European Food Safety Authority (EFSA) [51]. Comprehensive Antibiotic Resistance Database (CARD) Variants v4.0.0 [52], ARMFinder [49], and ResFinder v4.3.2 [53] were used to identify the antibiotic resistance genes (ARGs). BLASTN v2.8.1 + was used to detect virulence factors (VFs) by searching against the setB database from the Virulence Factor Database (VFDB) [54] and VirulenceFinder v2.0.3 [55]. BLASTP v2.8.1 + search was performed against the Pathogen Host Interaction v4.14 database to identify the probable pathogenic genes (PGs) in the M3R3 genome [54]. The probiotic potential risk score (PPRS) was computed as defined by Bai et al. [56] to evaluate the risks associated with M3R3 probiotic candidate. The score was classified as low-risk (≤ 4), medium-risk (4–6), and high-risk (≥ 6). All these analyses were implemented in ProbioMinServer [57].

Prophage regions and phage-associated genes were predicted using Phigaro v2.3.0 [58], whereas putative plasmids were identified using the PlasmidFinder database v2.1 (https://cge.food.dtu.dk/services/PlasmidFinder/, last accessed 1 December 2024), applying thresholds of ≥ 95% identity and ≥ 60% coverage [59]. CRISPR arrays and associated Cas proteins were annotated with CRISPRCasFinder v2.2 [60].

Mobile genetic elements were screened via BLASTX against the mobileOG-db v1.1.3 [61], applying cutoff of > 90% identity and > 90% coverage. Biosynthetic gene clusters were identified using antiSMASH 6.0 with default parameters [62].

### In Silico Search for *bsh* Gene Candidates

Putative *bsh* genes in the genome of strain M3R3 were identified using the BLASTp algorithm with *bsh1* of *Lactiplantibacillus plantarum* WCFS1 as the query (CCC80500). Seventy-eight protein sequences of *L. brevis* annotated as linear amide C-N hydrolase (PFAM: PF02275) were retrieved from NCBI RefSeq proteins database applying both sequence and domain cutoffs above 20.4 (last accessed March 2025). The sequences were collected into a local database, and two truncated proteins were manually removed, resulting in a dataset of 76 high-quality sequences. In addition, seven penicillin V acylases (PVA) proteins were included as reference sequences, comprising three from *L. plantarum* (Q88SP0, Q890F5, and Q88UC9), three from *L. brevis* (Q03PK6, Q03NN7, and Q03P51), and one from *Latilactobacillus sakei* (Q38Z70), as reported by O’Flaherty et al. [5].

All proteins sequences were aligned using the COBALT multiple sequence alignment tool [63]. Phylogenetic reconstruction was carried out with the NCBI Tree Viewer, using the Fast Minimum Evolution algorithm with the Grishin model for evolutionary distances calculation. The phylogenetic tree was visualized as described above.

### RT-PCR Assay

RNA isolation was carried out from M3R3 cells grown in the presence of 1% BSs mixture and under control condition (MRS medium), as previously reported by Solieri et al. [64]. Residual genomic DNA was eliminated by incubating 2 µg of total RNA with dsDNase (Cat. No. EN0771) at 37 °C for 2 min in a pre-warmed thermocycler set with the lid temperature at 37 °C. Samples were then placed on ice, briefly centrifuged, and 500 ng of the treated RNA were used for first-strand cDNA synthesis carried out using the RevertAid Reverse Transcriptase (Cat. No. EP0441) in presence of random hexamers (Cat. No. SO142) and oligo (dT)_18_ primers (Cat. No. SO131), according to the manufacturer’s recommendations. All reagents for RT were from Thermo Fisher Scientific (Waltham, MA, USA). *Pva* and *bsh*-targeted RT-PCR amplifications were carried out using the primer pairs listed in supplementary Table S2.

### Penicillin V MIC Determination

Antibiotic susceptibility to Penicillin V (PenV) was assessed using the broth microdilution method [65]. The Minimum Inhibitory Concentration (MIC) was determined according to the break-point values established for obligate heterofermentative lactobacilli (including *L. brevis*) [66], in order to classify the strain as resistant or susceptible to PenV, as recommended by EFSA [51].

### Statistical Analysis

All analyses were conducted in triplicate and results are expressed as mean values ± standard deviation (SD). Statistical analyses and graph generation were performed with GraphPad Prism v10.3.1 (GraphPad Software, La Jolla, CA, USA). Unless otherwise specified, data were analyzed using one-way ANOVA followed by Dunnett’s post hoc test. A *p*-value < 0.05 was considered statistically significant.

## Results and Discussion

Recently, several studies have underscored the pivotal role of BSH in lipid metabolism and in mediating host–microbe interactions [67–69]. Consequently, BSH activity is widely recognized as a key criterion for probiotic selection, and numerous investigations have examined the distribution of *bsh* genes across different species, correlating them with substrate-specific BSH activities [70]. In the present study, we employed a combined phenotypic and genomic approach to characterize *L. brevis* M3R3 as promising probiotic candidate. The strain, part of the microbial collection of Probioetna srl, was subjected to a series of probiotic assays, including a plate-based evaluation of BSH ability. Preliminary results revealed that M3R3 exhibited a marked capacity to deconjugate BSs (data not shown), thereby providing a strong rationale for further studies to validate its selection as a BSH-active candidate.

### Inhibitory Effect of Individual Bile Salts

As previously demonstrated, BSs tolerance was largely attributed to BSH-mediated deconjugation of BSs, which enhances cell viability under gastrointestinal conditions [6, 10]. To evaluate the impact of different concentrations of individual conjugated BSs on the growth of *L. brevis* strain M3R3, a stress response assay was performed by supplementing MRS medium with four individual BSs, each tested at four distinct concentrations. These concentrations were selected to reflect the range of physiological BSs concentrations in the small intestine under fed conditions (0.3 to 0.8%, *w/v*). The growth of M3R3 strain under each condition was assessed by plate counts relative to the control (MRS medium without BSs). The M3R3 strain displayed comparable tolerance to all four tested BSs with plate counts ranging from a minimum of 8.73 ± 0.06 to a maximum of 9.37 ± 0.08 Log_10_ CFU/mL (Table 1). These findings highlight the robust ability of M3R3 strain to grow in presence of various concentrations of conjugated BSs even exceeding the physiological concentration of BSs.

**Table 1:** Effects of different concentrations of individual BSs on the growth of *Levilactobacillus brevis* M3R3 strain. Log_10_ CFU/mL values were measured after 24 h of exposition to conjugated BSs relative to the control (MRS without BSs). Data are the mean of at least three replicates. Statistically significant differences compared with the control were determined by one-way ANOVA (*p* < 0.05) and are indicated with different letters. Abbreviations: TDCA (taurodeoxycholic acid), TCA (taurocholic acid), GDCA (glycodeoxycholic acid), GCA (glycocholic acid), and na, not applicable

Conditions	Concentrations (%)				
	0.0	0.1	0.25	0.5%	1.0%
MRS (CTRL)	9.45 ± 0.02^a^	na	na	na	na
TDCA	na	9.31 ± 0.02^a^	9.22 ± 0.30^a^	9.12 ± 0.12^a^	9.07 ± 0.10^b^
TCA	na	9.13 ± 0.02^b^	9.17 ± 0.12^a^	9.19 ± 0.15^a^	9.20 ± 0.22^a^
GDCA	na	9.27 ± 0.27^a^	9.27 ± 0.27^a^	9.21 ± 0.24^a^	8.98 ± 0.04^b^
GCA	na	9.37 ± 0.08^a^	9.30 ± 0.14^a^	8.87 ± 0.24^b^	8.73 ± 0.06^b^

Except for TCA, increasing concentrations of individual BSs generally corresponded to higher growth inhibition, indicating a dose-dependent effect on strain viability. The highest reduction in plate counts was detected in the presence of 1% and 0.5% GCA, followed by 1% GDCA. This finding is consistent with previous studies reporting that glyco-conjugated BSs exert a more pronounced inhibitory effect on lactobacilli growth than their tauro-conjugated counterparts [6, 71].

### Growth Kinetics and Deconjugation Activity under Exposure to Individual Conjugated Bile Salts

**Fig. 1:**
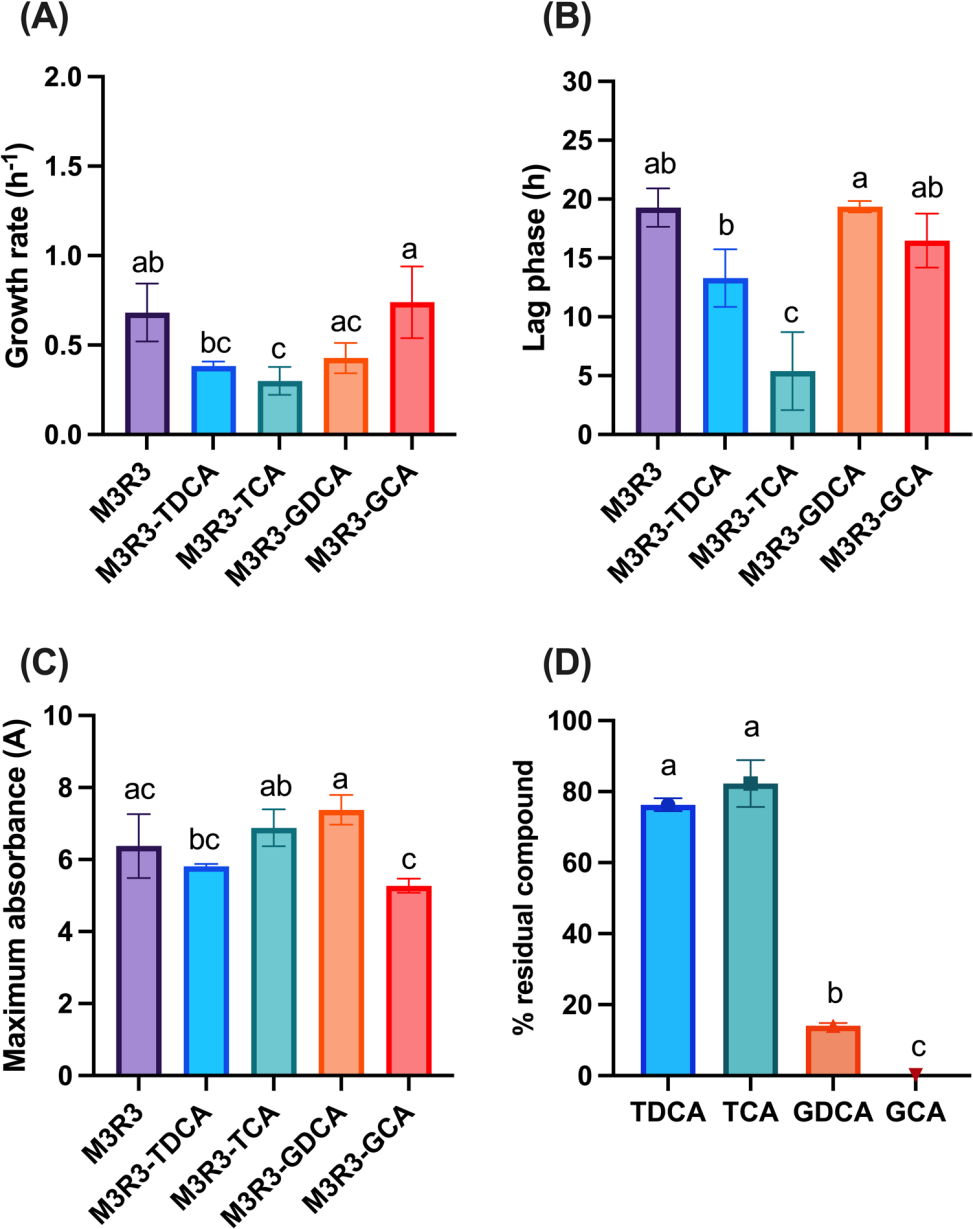
Growth kinetics parameters and bile salts deconjugation ability of *Levilactobacillus brevis* M3R3 in the presence of 1.0% (w/v) of individual bile salts (TDCA, TCA, GDCA and GCA) compared with the control condition (MRS medium)*.* Kinetics parameters*,* such as (**A**) specific growth rate (µ, h^−1^), (**B**) lag phase (λ, h), and (**C**) maximum cell density (A, OD_600nm_)*,* were estimated using Grofit package. (**D**) Deconjugation ability expressed as the percentage of residual conjugated bile salt (TDCA, TCA, GDCA, or GCA) quantified in cell-free supernatants collected during the late stationary phase. Significant differences were calculated with one-way ANOVA and indicated with different letters. Graphs are visualized with GraphPad Prism v10.3.1 software (San Diego, CA, USA). Abbreviations: TDCA, taurodeoxycholic acid; TCA, taurocholic acid; GDCA, glycodeoxycholic acid; and GCA, glycocholic acid

To further investigate the adaptive response of strain M3R3 to conjugated BSs and to correlate this response with its deconjugation activity, growth curves were determined in the presence of 1.0% of each individual BS. Growth curves (Fig. S1) were fitted using the three parametric models such as Gompertz, logistic and Richards equations implemented in the Grofit package. Based on AIC values, the Gompertz or logistic models generally provided the best fit to the experimental data, whereas the Richards equation consistently failed to describe the observed growth profiles (Table S3). In one case (1.0% TCA), the parametric models could not be applied, and data were instead fitted using the spline-based model, a non-parametric smoothing approach that uses smoothing splines to approximate the shape of the observed growth data without assuming a predefined mathematical model (Table S3). The derived kinetic parameters are summarized in Fig. 1.

It is important to note that, under TCA and GCA exposure, the number of sampling points during the lag and exponential phases was limited (Fig. S1). This scarcity of data in the most informative growth intervals reduces the reliability of the corresponding kinetic estimates and should therefore be interpreted with caution. Despite these limitations, exposure to individual conjugate BSs did not significantly affect the specific growth rate of M3R3 strain under GDCA, GCA and TDCA exposure, whereas TCA condition resulted in a significant reduction compared to the control condition (*p* < 0.05) (Fig. 1a). Similarly, the lag phase duration remained unchanged in the presence of GDCA and GCA but was significantly shortened under TDCA and TCA exposure compared to the control condition (*p* < 0.05), suggesting a possible stimulatory or priming effect, rather than inhibition (Fig. 1b). Maximum growth efficiency (A) remains unaffected by any of the tested BSs (Fig. 1c).

The deconjugation ability of M3R3 strain was evaluated by determining the percentage of residual conjugated BSs in the cell-free supernatant collected at stationary phase, using UHPLC/HR-MS. As shown in Fig. 1d, semi-quantitative AUC analysis revealed a marked difference in deconjugation efficiency between glyco-conjugated and tauro-conjugated BSs. Strain M3R3 efficiently deconjugated GDCA and GCA, leaving residual concentrations of 14.2% and 0.1%, respectively. By contrast, deconjugation of tauro-conjugated BSs was markedly less efficient, with 83.4% TDCA and 76.3% TCA remaining.

### Growth Kinetics and Deconjugation Activity Under Bile Salt Mixture Exposure

**Fig. 2:**
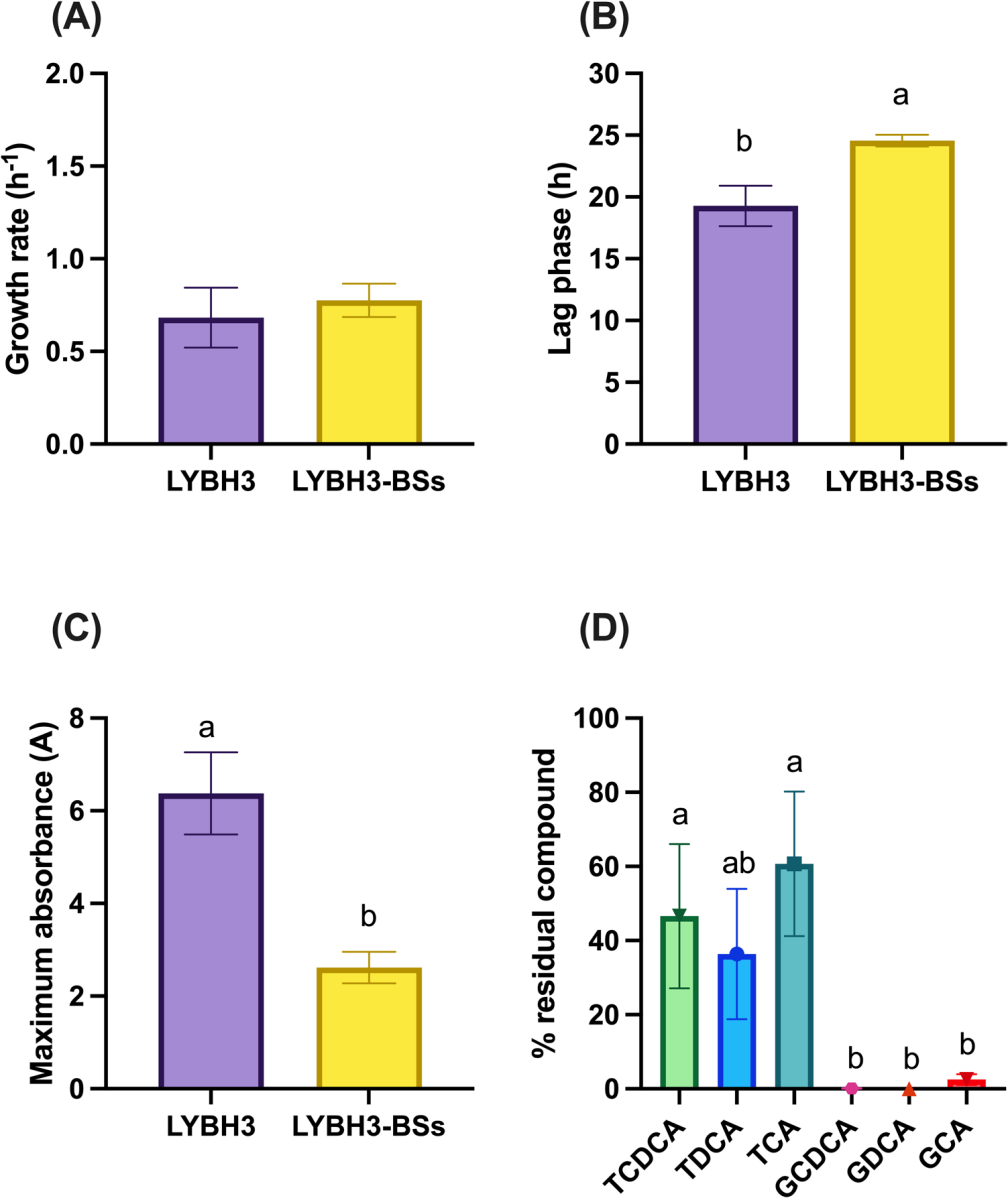
Growth kinetics parameters and bile salts deconjugation ability of *Levilactobacillus brevis* M3R3 in the presence of 1.0% (w/v) of bile salts (BSs) mixture compared with the control condition (MRS medium)*.* Kinetics parameters, such as (**A**) specific growth rate (µ, h^−1^), (**B**) lag phase (λ, h), and (**C**) maximum cell density (A, OD_600nm_), were estimated using Grofit package. Significant differences in growth kinetics parameters were calculated between control and stress conditions with a two-tailed *t*-test and indicated as follows: **, *p* ≤ 0.01. (**D**) Deconjugation ability expressed as the percentage of residual conjugated bile salt (TCDCA, TDCA, TCA, GCDCA, GDCA, or GCA) quantified in cell-free supernatants collected during the late stationary phase. Significant differences among residual compound percentages were calculated with one-way ANOVA and indicated with different letters. Graphs are visualized with GraphPad Prism v10.3.1 software (San Diego, CA, USA). Abbreviations: TCDCA, taurochenodeoxycholic acid TDCA, taurodeoxycholic acid; TCA, taurocholic acid; GCDCA, glycochenodeoxycholic acid; GDCA, glycodeoxycholic acid; and GCA, glycocholic acid

The survival and deconjugation activity of M3R3 strain were further assessed in the presence of BSs mixture, a condition which more closely mimics the complex in vivo bile environment compared to individual BSs exposure [31, 72]. The resulting growth curves (Fig. S2) were modeled to determine the kinetic parameters μ, A, and λ (Table S3). As shown in Fig. 2a, exposure to the BSs mixture did not significantly affect the specific growth rate, consistent with the results obtained using individual BSs (Fig. 1a). In contrast, the BSs mixture significantly increased the lag phase duration (*p* < 0.01) (Fig. 2b), supporting a delayed adaptation to the complex bile environment. This effect contrasts with the response observed under exposure to TDCA and TCA alone (Fig. 1b), where the lag phase was shortened. Furthermore, a significant decrease in A value was observed under BSs mixture stress compared to the control (*p* < 0.01), indicating that the biomass production is markedly inhibited under this condition (Fig. 2c). A study conducted by Hu and co-workers [72] characterized commercial BSs mixture powders and demonstrated the presence of several other BSs beyond TDCA, TCA, GDCA, and GCA, which can impair the growth of strain M3R3 more severely than the individual BSs. Furthermore, it can be speculated that, unlike the in vivo gut environment, the experimental system used here is closed and lacks efflux mechanisms, leading to the accumulation of deconjugated BSs. Such accumulation may reduce cell viability and contribute to the delayed adaptation observed in strain M3R3, thereby potentially overestimating its sensitivity to BSs mixture. Consistently, a recent study revealed that, contrary to the earlier assumptions, certain deconjugated BSs can exert more toxic effects on bacterial cells than their conjugated counterparts [73].

The semi-quantitative analysis of residual conjugated BSs revealed a marked variability in BSH activity of M3R3 strain related to the substrate. Notably, GCDCA and GDCA were completely deconjugated, while GCA exhibited a residual compound of 2.5%. Among tauro-conjugated BSs, TDCA displayed the lowest residual percentage (Fig. 2d). By contrast, M3R3 strain displayed the lowest efficiency in deconjugating TCDCA and TCA, which persisted in their conjugated forms, showing a residual percentage of 46.6% and 60.7% respectively (Fig. 2d).

Collectively, the BSs deconjugation assays in presence of both individual BSs and BSs mixture supported that the M3R3 strain exhibits a higher deconjugation capacity toward glyco-conjugated BSs compared to tauro-conjugated forms, suggesting the presence of BSH activity under the tested conditions. Notably, BSH-positive lactobacilli strains have been reported to have a preferential deconjugation activity towards glyco-conjugated BSs, which are more cytotoxic than tauro-conjugated counterparts [6, 71]. Glycine preference could reflect the increased abundance of glycine-conjugated BSs among vertebrates, whereas taurine specificity is restricted to only a few related BSHs [73]. This trend was consistently observed in all experimental conditions, with the most pronounced deconjugation observed in M3R3 exposed to the BSs mixture, reflecting a scenario that more closely resembles physiological conditions.

### General Genomic Features and Taxonomic Evaluation

**Fig. 3:**
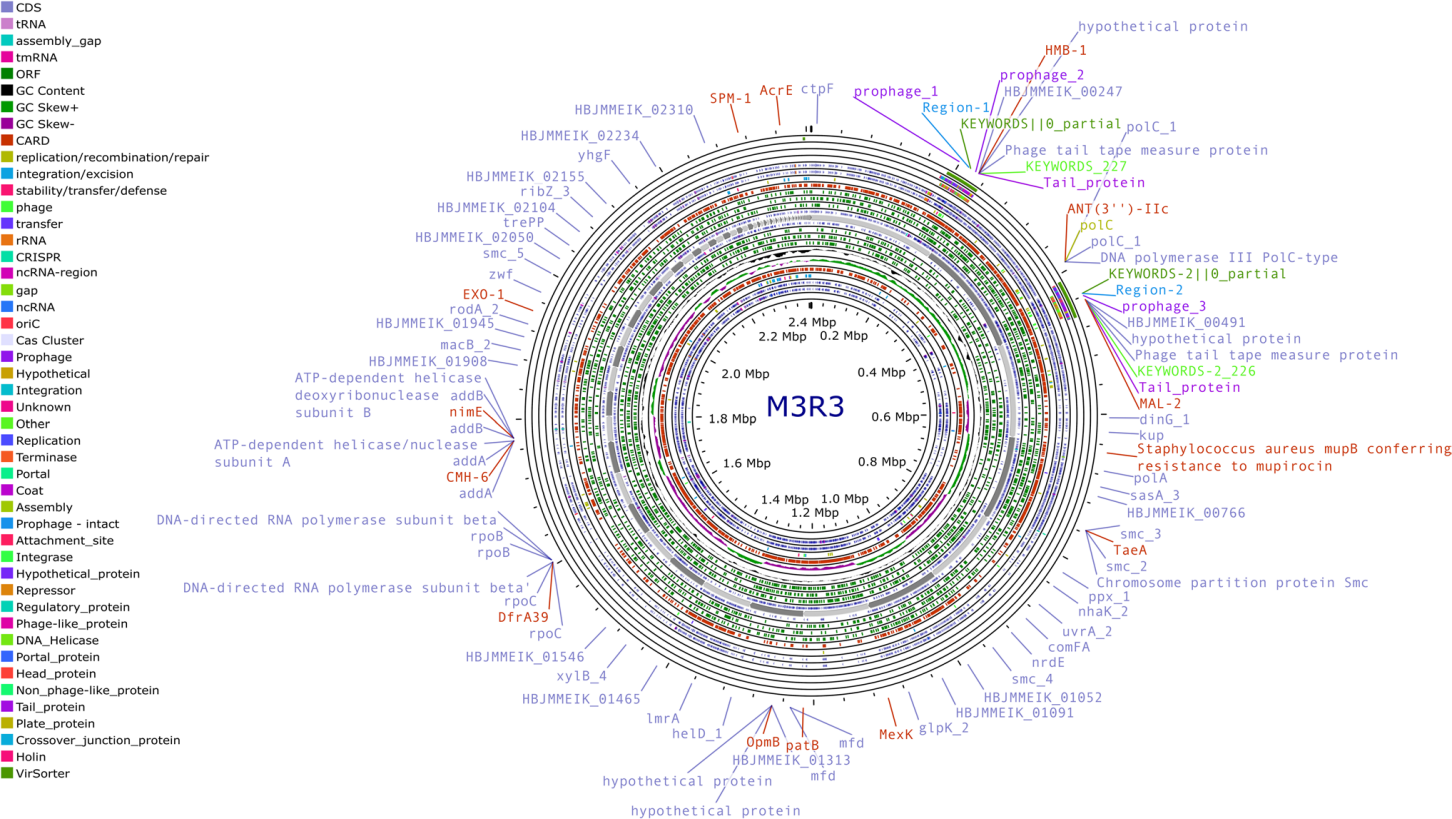
Circular graphical representations of the *Levilactobacillus brevis* M3R3 strain contigs obtained using Proksee (https://proksee.ca/; last accessed 13 February 2025). Starting from the outermost ring: Ring 1: PHASTEST, Ring 2: Phigaro, Ring 3: CRISPRCasFinder Annotation (+), Ring 4: Bakta Annotation (+), Ring 5: Prokka Annotation (+), Ring 6: mobileOG-db Annotation (+), Ring 7: CARD RGI Results (+), Rings 8 to 10: ORFs (+ 3, + 2, + 1), Ring 11: Features (+), Backbone (Contigs), Ring 13: Features (-), Rings 14 to 16: ORFs (−1,−2,−3), Ring 17: GC Content, Ring 18: GC Skew, Ring 19: CARD RGI Results (-), Ring 20: mobileOG-db Annotation (-), Ring 21: Prokka Annotation (-), Ring 22: Bakta Annotation (-), Ring 23: CRISPRCasFinder Annotation (-)

To investigate the genetic determinants underlying the BSH-positive phenotype of *L. brevis* strain M3R3 and to evaluate its probiotic potential, whole-genome sequencing was performed. Reads assembly resulted in 72 contigs, corresponding to a total genome length of 2,406,595 bp (Fig. 3). The GC content was 45.93% and the orientation of positive and negative strands was clearly defined.

Genome annotation predicted a total of 2442 genes in the M3R3 genome assembly, including 2373 CDS, 66 tRNA, 6 rRNA, and 1 tmRNA (Table 2). The functional annotation by BLASTKOALA assigned approximately 50.08% of these CDS (1207 genes) into 23 different functional KEGG categories. The most represented categories were the followings: protein families: genetic information processing (190, 15.73%), genetic information processing (156, 12.91%), protein families: signaling and cellular processes (142, 11.75%), and carbohydrate metabolism (125, 10.35%) (Fig. S3). 50.02% of CDS were assigned to an unknown function. A plasmid replicon sequence, rep28(pCIS4), was identified in the M3R3 genome with 99.78% identity and 100% query coverage, indicating the presence of a plasmid homologous to the *Lactococcus lactis* subsp. *cremoris* UC509.9 plasmid pCIS4 (NCBI accession: CP003162.1). A similar plasmid was found in the probiotic candidate *L. brevis* strain H3 isolated from a traditional fermented beverage in India [18]. Two CRISPR arrays and one ORF encoding a Cas4-type II protein were detected in the genome of strain M3R3. However, no CRISPR arrays were found in proximity to this ORF (Table S4).

**Table 2:** Assembly and annotation of *Levilactobacillus brevis* M3R3 genome

Assembly statics	Features	Annotation statics	Features
Contigs	72	CDS	2373
Contigs (> = 1000 bp)	65	tRNA	66
Contigs (> = 5000 bp)	41	tmRNA	1
Contigs (> = 10,000 bp)	34	rRNA	6
Contigs (> = 25,000 bp)	24	ncRNA	6
Contigs (> = 50,000 bp)	14	ncRNA Region	22
Largest contig	239,718	sORF	0
Total length	2,406,595	Gap	2
GC (%)	45.93	oriC	2
N50	124,627	oriV	0
N90	24,367	oriT	0
auN	118,405.8		
L50	7		
L90	25		
Properly paired (%)	99.3		
Avg. coverage depth	117		
Coverage > = 1x (%)	99.99		

**Fig. 4:**
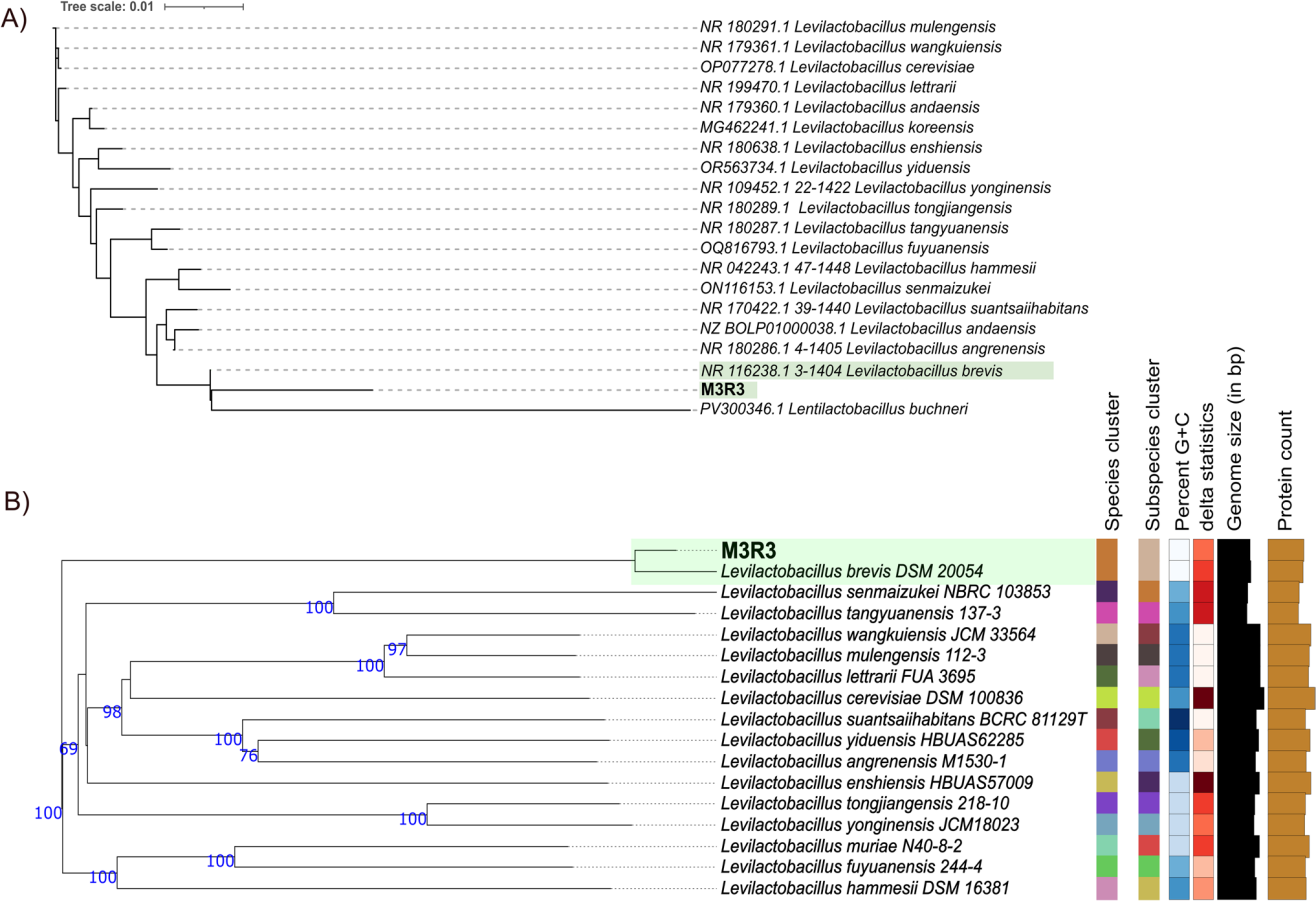
Phylogenetic analyses inferring the taxonomical position of *Levilactobacillus brevis* M3R3. **(A)** 16S rRNA gene-based phylogeny constructed using Neighbor-Joining (NJ) algorithm. **(B)** Phylogenomic tree constructed using GBDP distances derived from the genomes of TYGS type strains most closely related to *L. brevis* M3R3

Phylogenetic analysis based on 16S rRNA gene sequences and genome-based taxonomic analysis using the TYGS web server revealed that *L. brevis* strain M3R3 clustered with the *L. brevis* reference strain and was clearly distinguished from closely related species (Fig. 4). Accordingly, the average nucleotide identity (ANI) and dDDH values between strain M3R3 and *L. brevis* DSM 20054^ T^ were 97.73% and of 79.4%, respectively, both exceeding the accepted thresholds for species delineation (Tables S5 and S6). The results confirm that strain M3R3 indeed belongs to the species *L. brevis*.

### Comparative Genomics

**Fig. 5:**
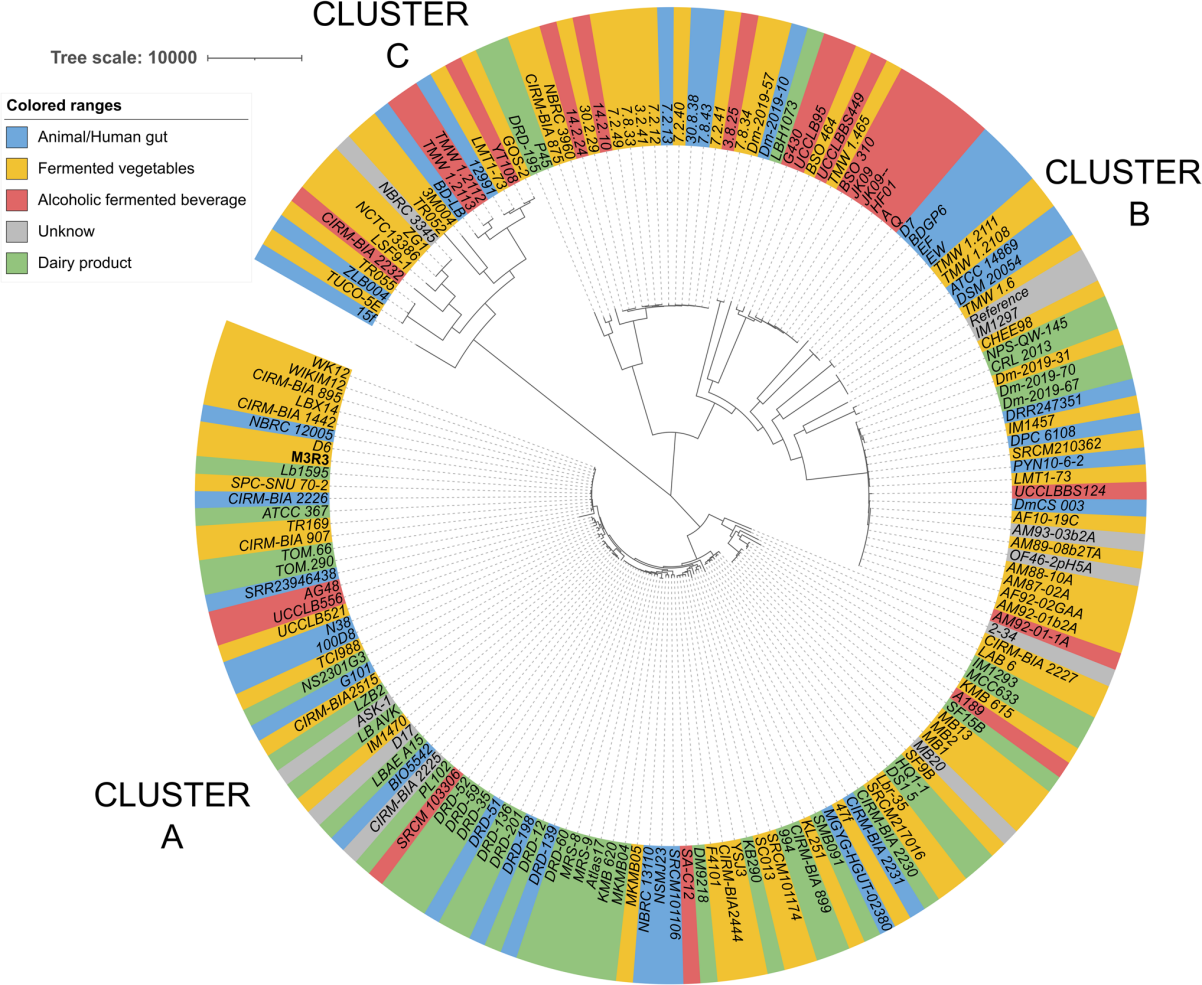
Maximum-likelihood phylogenetic tree of 175 *Levilactobacillus brevis* strains built on core SNP alignments (3059 SNP found with snippy multi v4.6.0). Branch colors indicate isolation sources: yellow, fermented vegetables; light blue, animal and human gut; red, alcoholic fermented beverages; green, dairy products; grey, unknown, respectively. Clusters A, B, and C are indicated. The tree was visualized using iTOL v7 [50]

The genome of strain M3R3 was compared with 174 publicly available *L. brevis* genomes deposited in the NCBI database. These genomes were categorized according to four major isolation sources: alcohol fermentation (25 strains, 14.4%), animal/human gut (38 strains, 21.8%), dairy products (35 strains, 20.1%), and fermented vegetables (64 strains, 36.8%). The remaining 6.9% were unknown. The resulting phylogenetic tree revealed the presence of three distinct *L. brevis* lineages, designated as cluster A, B, and C (Fig. 5). Although the clustering of strains did not perfectly correlate with their isolation sources, strains from alcoholic fermentation and from animal/human gut were mainly associated with cluster A. In contrast, the majority of strains isolated from dairy products (85.7%) and fermented vegetables (56.9%) were grouped in cluster B, which included also strain M3R3, whereas cluster C comprised a small number of strains with diverse origins. These findings supported a high degree of genomic diversity and potential functional adaptation within the species and are consistent with previous studies describing *L. brevis* as a genetically diverse species possessing a remarkably large pan-genome [74, 75].

**Fig. 6:**
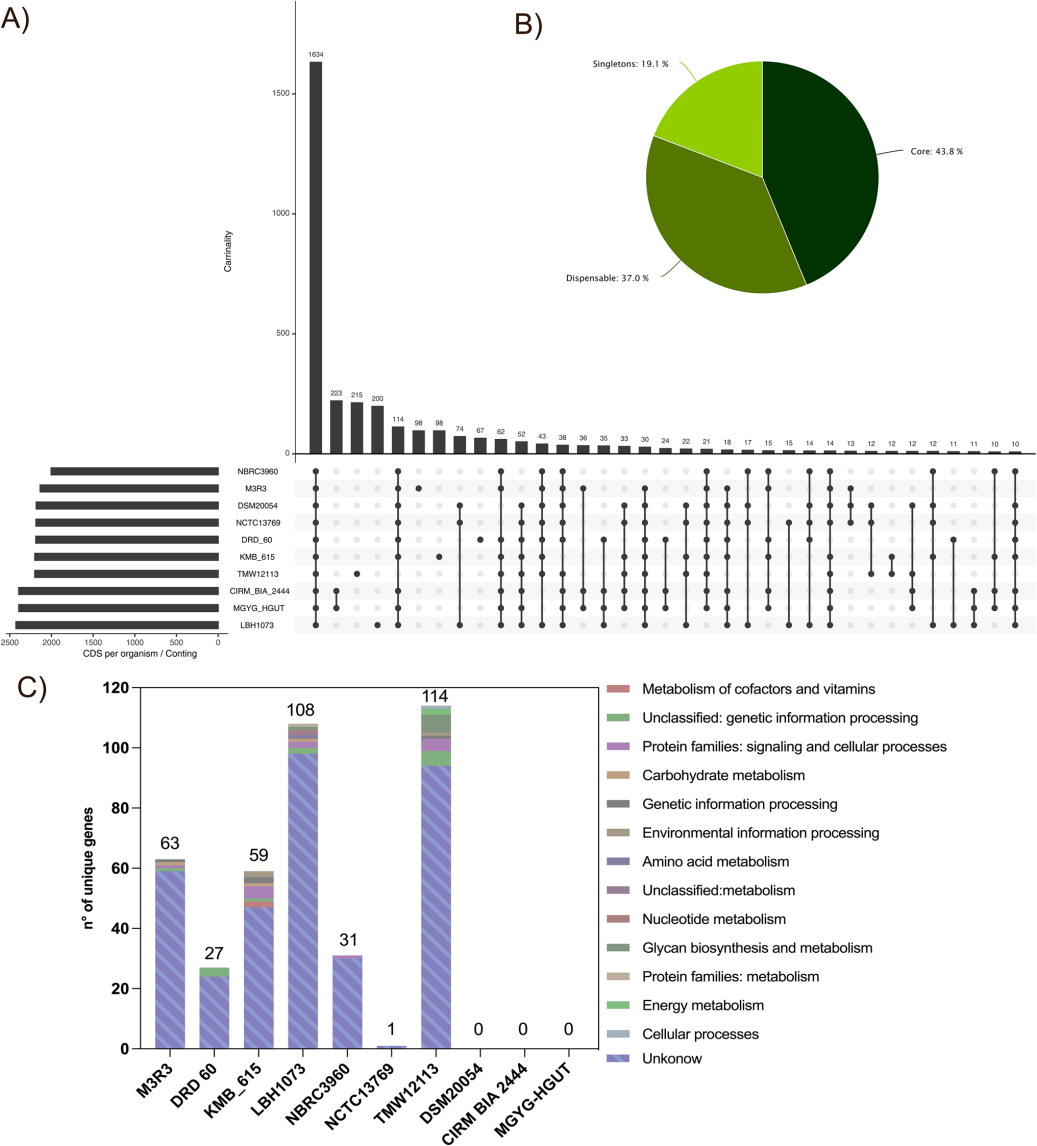
Comparative genomic analysis of *Levilactobacillusbrevis* M3R3 and nine conspecific strains. **(a)** Upset plot showing shared orthogroups of *L. brevis* strains (n = 10). **(b)** Percentage distribution of core, dispensable, singleton genes. **(c)** KEGG Orthology (KO) annotation of singletons across the ten *L. brevis* genomes

A comparative genome analysis was carried out on a subset of 10 strains representative of the three distinct clusters previously described. The pan-genome comprised 4,288 COGs, with 36.3% classified as core genes and 32.9% as strain-specific genes. Non-essential genes, defined as those absent in at least one strain, accounted for 30.8% (Fig. 6a and b). Together with the strain-specific genes, these constituted the accessory genome. These findings are consistent with the high plasticity of the *L. brevis* genome, which tends to adapt its features depending on the environment [74, 75]. This is also reflected in the relatively low percentage of core genes shared among the ten *L. brevis* strains, which falls below 50%.

The distribution of strain-specific clusters revealed that strain M3R3 differed from other *L. brevis* strains in the number of singleton genes. Specifically, 63 genes were uniquely found in the M3R3 genome. Among all strains, *L. brevis* TMW 1.2113 had the highest number of unique genes (114), whereas the NTC13768 genome had the fewest, with only 1 singleton (Fig. 6c). Based on KEGG Orthology (KO) annotation, the singletons of strain M3R3 were predominantly classified as hypothetical proteins (93.65%), with only four genes assigned to functional categories, including genetic information processing, signaling and cellular processes, carbohydrate metabolism, and genetic information processing (Fig. 6c).

### Biosafety Assessment and Genome Stability Evaluation

CARD and ARMFinder analyses did not reveal any ARGs, indicating that strain M3R3 can be considered safe in relation to the potential dissemination of antibiotic resistances. In contrast, ResFinder analysis identified the *ClpL* gene as a potential antimicrobial resistance gene (Table S7). However, in *Lactobacillaceae* family, this gene encodes an ATP-dependent Clp protease with chaperone activity which is involved in degrading misfolded or damaged intracellular proteins [76]. Clp is inducible under acidic shock [77] and contributes to the fast response of lactobacilli to harsh gastro-intestinal conditions, especially to acid and bile stresses [76].

Search for pathogenic genes did not find any candidates, except for the *WalR* gene, which a member of one of the two-component systems involved in sensing and reacting to environmental changes (Table S7). The *walR* regulates cell wall metabolism, ensuring structural integrity and adaptability in harsh environments such as the gastrointestinal tract [78]. The M3R3 genome displayed the *WalK* gene upstream to *WalR*, suggesting that the two-component system is complete. The same two-components system was found in a *L. brevis* GABA-producing probiotic candidate [79]. According to its safe status, the associated PPRS, calculated by ProbioMin, was 1.00 for strain M3R3 (low risk) (Table S7).

Three prophage regions were identified in the M3R3 genome, one belonging to *Siphoviridae* and the other two belonging to *Myoviridae* family (Table S8). *Siphoviridae* and *Myoviridae* are prophages frequently detected in *L. brevis* genomes [74]. Furthermore, the genome of strain M3R3 accounted for 140 mobile elements, most of them were associated with integration/excision (47) and replication/recombination/repair (44) (Fig. S4).

### Search for Probiotic-Related Functional Traits

**Fig. 7:**
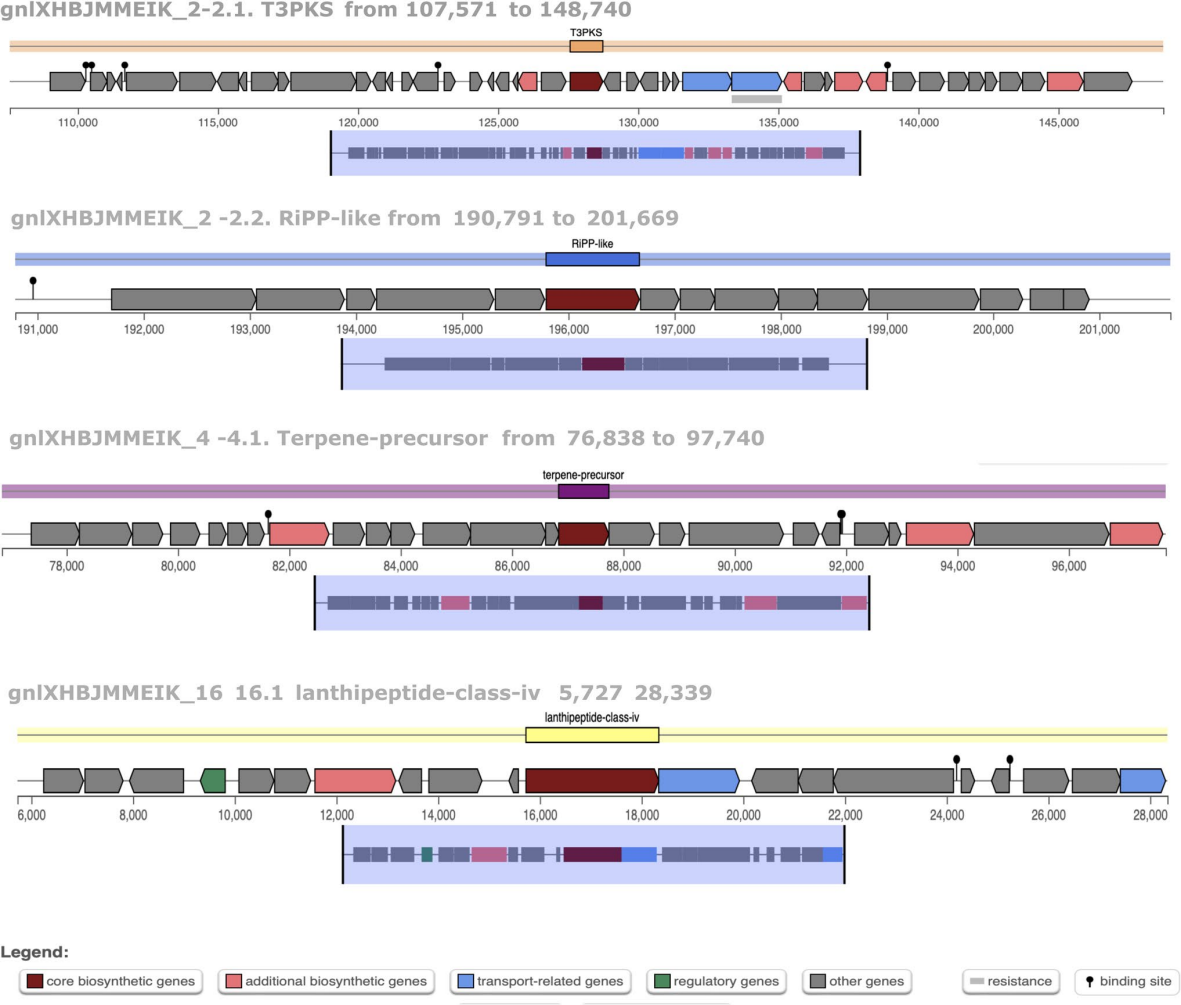
Biosynthetic gene clusters (BGCs) in the genome of *Levilactobacillus brevis* M3R3 predicted by antiSMASH. Candidate clusters were stringently filtered and grouped into families. Arrows are color-coded according to the enzyme family of the predicted product, with gene annotations shown below each arrow. One candidate cluster overlapped with or showed similarity to ribosomally synthesized and post-translationally modified peptide (RiPP) BGCs, while the others clusters included a type III polyketide synthase (T3PKS) region, a terpene precursor region, and a class IV lanthipeptide cluster

AntiSMASH analysis revealed that the genome of strain M3R3 contained four BGCs encoding lanthipeptide class IV, Type III polyketide synthase (T3PKS), a ribosomally synthesized, post-translationally modified peptide (RiPP-like compound), and terpen precursors (Fig. 7). Three of them matched with experimentally validated BGCs present in MIBiG 2.0 repository (Table S9). All the BGCs were predicted to encode antimicrobials or enzymes involved in the synthesis of antimicrobials. In detail, class IV lanthipeptides are characterized by multiple lanthionine rings and exert strong antimicrobial activity against several Gram-positive bacteria, including *Streptococcus* spp, *Clostridium difficile*, and *Bacillus* spp. [80, 81]. RiPPs encompass a broad category of natural products derived from ribosomal synthesis followed by enzymatic modifications. The detection of RiPP-like regions suggests the presence of additional pathways for producing bioactive peptides that could function as antimicrobials or signaling molecules (Fig. 7). Type III polyketide synthases (T3PKS) are involved in the biosynthesis of polyketides, which are secondary metabolites with diverse biological activities, including antimicrobial, antifungal, and anticancer properties [82]. Lactobacilli-derived terpenes have been recently demonstrated to inhibit efflux pumps in drug-resistant pathogens [83].

### Identification of *bsh* Gene Candidates and RT-PCR Assay

To identify putative *bsh* genes responsible for the previously observed BSs-deconjugation activity, the genome sequence of strain M3R3 was screened for linear amide C-N hydrolase proteins that encompass choloylglycine hydrolase (conjugated bile acid hydrolase, CBAH, EC:3.5.1.24), penicillin amidase (EC:3.5.1.11), and acid ceramidase (EC:3.5.1.23). The analysis identified 3 putative candidates, classified as *bsh_1A* (984 nt; 327 amino acids), *bsh_2A*, (978 nt; 325 amino acids), and *bsh_3A* (957 bp; 318 amino acids). BLASTp search against RefSeq NCBI protein database revealed that the deduced amino acid protein of *bsh_1A* was 100% identical to the choloylglycine hydrolase family protein WP_011668641.1, which displayed the Ntn_PVA domain (cd00542), described as Penicillin V acylase (PVA) (*E*-value: 5.13e-138). The amino acid sequences of *bsh_2A* and *bsh_3A* were 100% identical to the linear amide C-N hydrolases WP_011668417.1 and WP_011668908.1, respectively. These proteins possessed the conserve nnt_hydrolase domain (cI00467), which is present in the diverse superfamily of NtN hydrolases (*E*-value: 4.26e-71).

**Fig. 8:**
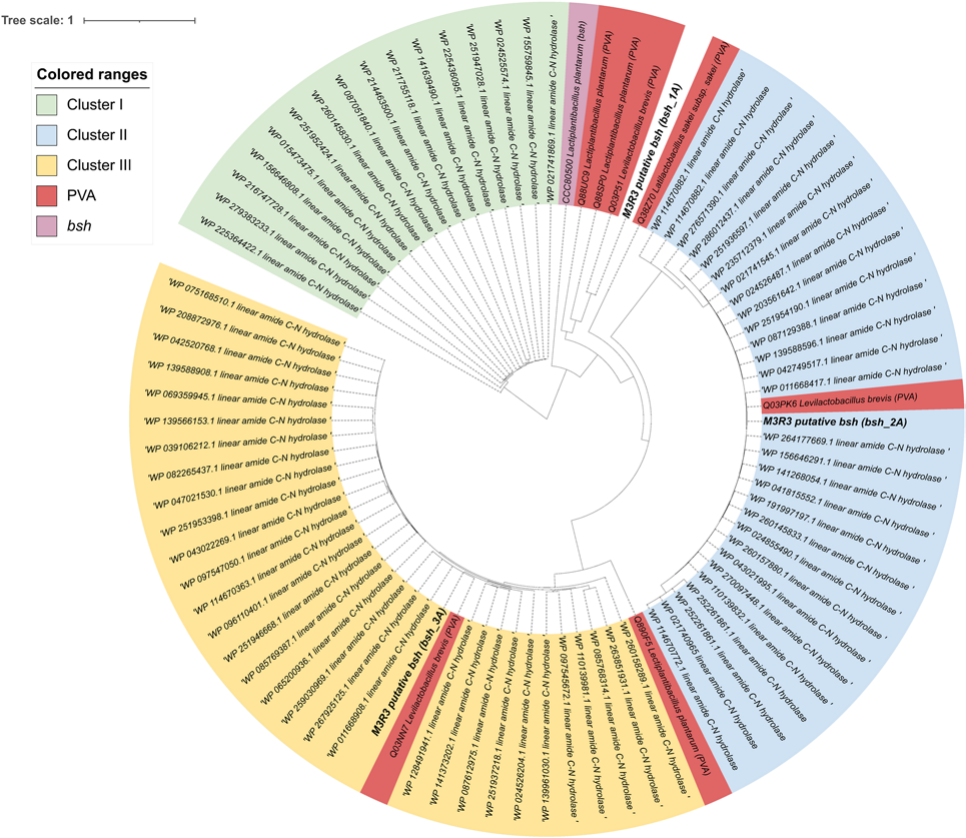
Fast minimum evolution tree of 79 putative linear amide C-N hydrolase proteins from *Levilacactobacillus brevis*. Seventy-six linear amide C-N hydrolase proteins of *L. brevis* were retrieved from RefSeq proteins in GenBank, whereas three linear amide C-N hydrolase proteins (in bold) were found in *L. brevis* M3R3 genome. Seven PVA (red) and one BSH (purple) proteins were used as control. Sequences were aligned using the COBALT tool with default parameters, while Grishin model was used to infer evolutionary distances. Labels represent protein ID. Clusters I, II, and III are depicted in green, blue, and yellow, respectively. The image was created using the interactive tree of life (iTOL) [50]

BSH and PVA are evolutionary related enzymes with high similarity in terms of the amino acid residues involved in their active site [5, 84]. This similarity makes it challenging to distinguish between the two proteins. The role of BSH in deconjugation of BSs is well established, whereas PVA appears to be involved in the degradation of penicillin-class antibiotics [85]. To further investigate whether the candidate genes found in M3R3 genome were truly *bsh* genes, the putative BSH proteins of M3R3 were aligned against 76 *L. brevis* RefSeq proteins annotated as amide C-N hydrolases in the NCBI database using the COBALT alignment tool. Seven representative PVA proteins and one representative BSH protein from *L. plantarum* WCFS1 (*bsh1*) were also included as controls. Based on the topology of the tree, the amide C-N hydrolases of *L. brevis* grouped into three distinct clusters, classified as I, II, and III (Fig. 8). Cluster I included 16 Protein IDs with an average length of 335.2 aa ± 29.4; cluster II included 29 Protein IDs (average length 325 ± 1.4 aa); and cluster III consisted of 31 Protein IDs with an average length of 316.8 ± 6.8 aa. These results were consistent with previous studies that demonstrated that putative *L. brevis bsh* genes clustered into three distinct phylotypes [9]. Notable, the majority of PVAs placed outside the clusters at the intersection between cluster I and cluster II. The putative BSH proteins identified in M3R3 genome were distributed differently across the clusters: bsh_2A and bsh_3A are located into the clusters II and III, respectively, whereas bsh_1A clustered away from other *bsh* genes and was closely related to PVAs (Fig. 8). Based on these findings, we concluded that the genome of *L. brevis* M3R3 possesses two *bsh* genes, namely *bsh_2A* and *bsh_3A*, and one putative PVA-encoding gene (previously referred to as *bsh_1A*, now re-called *pva*). Remarkably, no genes encoding BSH proteins from cluster I were found in the M3R3 genome.

**Fig. 9:**
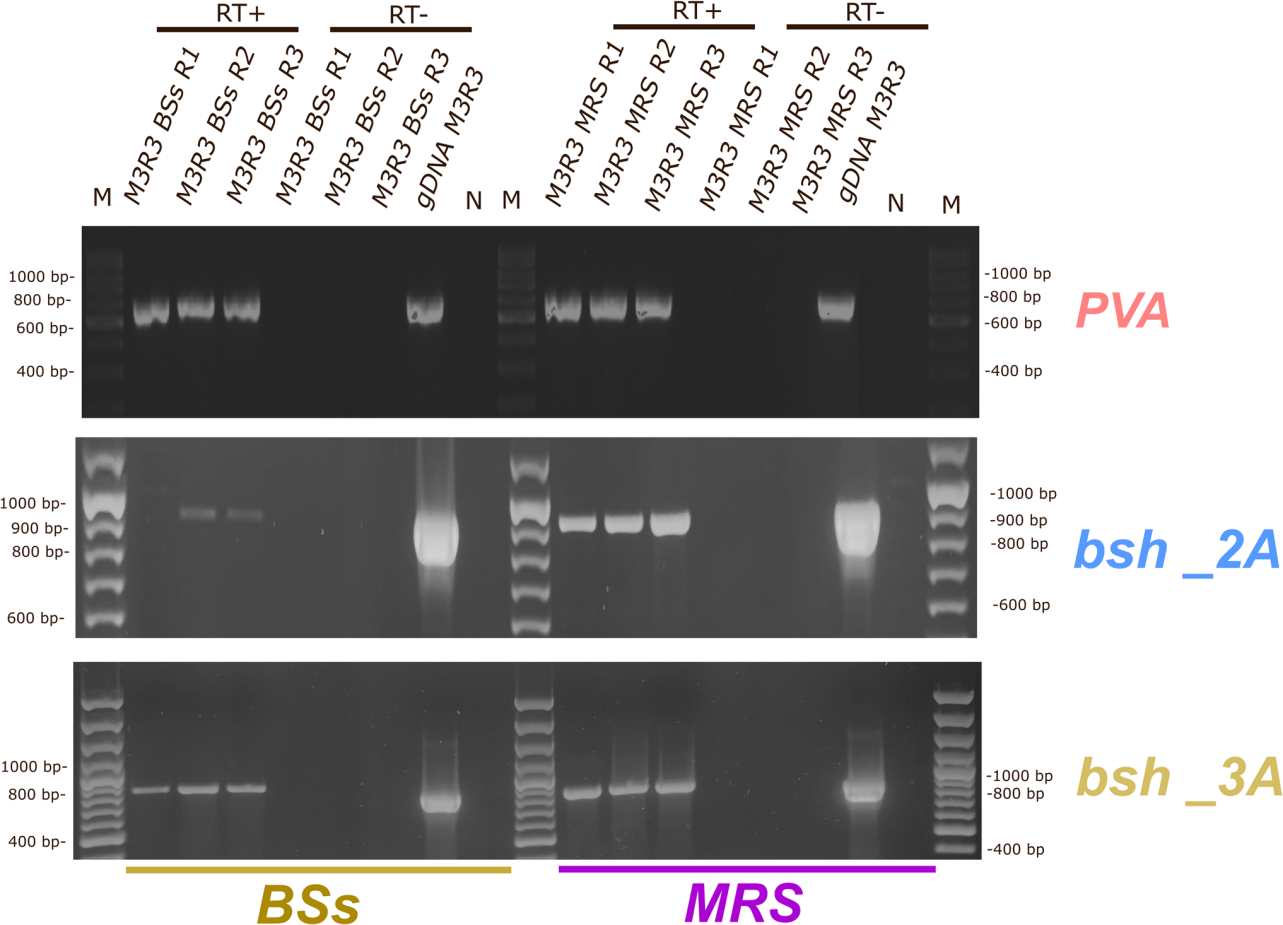
RT-PCR assay targeting the *pva*, *bsh_2A*, and *bsh_3A* genes in *Levilactobacillus brevis* M3R3 strain grown on MRS medium supplemented with 1% (*w/v*) BSs mixtures. MRS medium without any supplementation served as the control. The figure depicts amplified cDNAs; gDNA amplification was used as a positive PCR control. Abbreviations—M: molecular weight marker; N: negative control

To evaluate whether strain M3R3 modulates the expression of these genes in response to BSs mixture, we performed a RT-PCR assay targeting *pva*, *bsh_2A* and *bsh_3A*. As shown in Fig. 9, strain M3R3 actively transcribed all three target genes under both BSs stress and control conditions at stationary phase. These findings suggest that the genes are actively transcribed at the stage where the BSH activity has been evaluated and that the genes may be constitutively expressed or, alternatively, that their transcriptional regulation occurs at growth phases other than the stationary phase examined in this study. This result is also consistent with previous works indicating that exposure to bile does not necessarily induce *bsh* gene expression in BSH-active lactobacilli strains [73, 86]. Nevertheless, expanding the sampling strategy to earlier growth phases, particularly the early and the mid-exponential stages, would provide a more comprehensive understanding of *bsh* genes regulation in response to BSs-induced stress in strain M3R3. Furthermore, N-terminal autocatalysis and oligomeric assembly in homotetramers are critical post-translational modifications to ensure active BSH enzymes [87]. Therefore, analysis of protein patterns will be also necessary to definitively link one or more of these two genes to the observed BSH activity in M3R3.

### Antibiotic Susceptibility Test

Recently, PVA enzymes have been widely used in pharmaceutics to produce the 6-aminopenicillanic acid (6-APA), a key intermediate in the synthesis of semi-synthetic antibiotics [88]. Therefore, the presence of PVA in M3R3 could be considered a relevant genetic trait for future biotechnological applications. Although, PVA may also hydrolyze PenV, conferring resistance to this antibiotic [85]. To exclude that this gene can contribute to PenV antibiotic resistance, strain M3R3 was subjected to antibiotic susceptibility test against PenV. Strain M3R3 showed a MIC < 0.25 µg/mL. According to the EFSA breakpoints for obligately heterofermentative lactobacilli, this value indicates that the strain is sensible to PenV.

## Conclusion

BSH-active probiotics are increasingly recognized as valuable tools to modulate BSH activity in the human gut and promote host health. Studies in animal models showed that increasing BSH activity in the gut ecosystem can lead to reduced body weight gain, lower adiposity, and decreased levels of circulating Low Density Lipoprotein (LDL) cholesterol and triglycerides [69]. A recent study reported that non-obese liver fibrotic individuals displayed a significantly lower copy number of BSH encoding genes in their gut microbiome [89]. In the present study, the probiotic candidate M3R3, ascribed to the *L. brevis* species, demonstrated a robust tolerance to BSs, with a strong adaptive response when exposed both to a BSs mixture and to the individual BSs. Remarkably, the strain M3R3 showed a consistent capacity to deconjugate BSs, with a marked preference for glyco-conjugated forms, which are known to exert higher cytotoxicity the tauro-conjugated forms. Whole-genome analysis and comparative genomics identified two *bsh* genes, *bsh_2*A and *bsh_3A*, which are actively transcribed under BSs stress and under control condition and are likely responsible for the observed deconjugation activity of *L. brevis* M3R3. Although a *pva* gene was also detected, it did not confer resistance to PenV. The absence of ARGs and virulence determinants further support the safe profile for probiotic use of *L. brevis* strain M3R3. Additionally, the presence of BGCs with antimicrobial activity further emphasizes the potential of M3R3 as a multifunctional probiotic candidate.

Collectively, these findings underscore the relevance of *L. brevis* M3R3 as BSH-active, bile tolerant and genomically safe strain, with potential applications as probiotic supplement aimed at supporting lipid homeostasis and gut health. This study also reinforces the emerging role of non-intestinal lactobacilli in targeted modulation of the bile acid pool and cholesterol homeostasis.

## Supplementary Information

Below is the link to the electronic supplementary material.

ESM 1 (XLXS 636 KB)

ESM 2 (DOCX 568 KB)

## Data Availability

Sequence data that support the findings of this study have been deposited in the GenBank database with the accession codes PV902533 and PRJNA1293146.
