# Supplementary material for: Bile Salt Hydrolase Activity in the Food-Derived Strain Levilactobacillus brevis M3R3: Genomic and Functional Characterization
Source: Probiotics Antimicrob Proteins. 2025 Nov 19;18(4):5688–705. doi: 10.1007/s12602-025-10810-0 (PMC13342217; doi:10.1007/s12602-025-10810-0)
Supplement: Supplementary file 2 — (DOCX 568 KB) [file 12602_2025_10810_MOESM2_ESM.docx]

**Bile Salt Hydrolase Activity in the Food-Derived Strain *Levilactobacillus brevis* M3R3: Genomic and Functional Characterization**

Gianluigi Agolino^1,†^ Marianna Cristofolini^2,†^ Maria Anna Ronsivalle^2^, Alice Cattivelli^2^, Davide Tagliazucchi^2^, Cinzia Caggia^1,3^, Lisa Solieri^2,*^, Cinzia L. Randazzo^1,3^

^1^Department of Agricultural, Food and Environment, University of Catania, 100, 95123 Catania, Italy

^2^Department od Life Sciences, University of Modena and Reggio Emilia, 42122 Reggio Emilia, Italy

^3^Probioetna SRL, Spin off of University of Catania, via S. Sofia 100, 95123 Catania, Italy

^*^Corresponding author: Lisa Solieri, via Amendola 2, Besta Building, 42122 Reggio Emilia, Italy; phone number: +39 0522 522026; email address: [lisa.solieri@unimore.it](mailto:lisa.solieri@unimore.it)

^†^These authors contributed equally to this work

**Supplementary Figures**

**Fig. S1** Representative growth kinetics of *Levilactobacillus brevis* strain M3R3 in MRS medium supplemented with 1.0% (w/v) of each individual bile salt (BS), compared with the control condition (MRS only). Optical density at 600 nm (OD₆₀₀) was recorded over time. Each condition was tested in three independent biological replicates. Growth curves were modeled using the Grofit package in the R environment, applying both parametric (black) and spline-based (red) methods. Black and red dotted lines indicate the intercepts corresponding to the maximum growth rate (h⁻¹) calculated by the parametric and spline-based approaches, respectively. The vertical green line marks the sampling point used for supernatant collection for UHPLC/HR-MS analysis. Abbreviations: TDCA, taurodeoxycholic acid; TCA, taurocholic acid; GDCA, glycodeoxycholic acid; GCA, glycocholic acid.


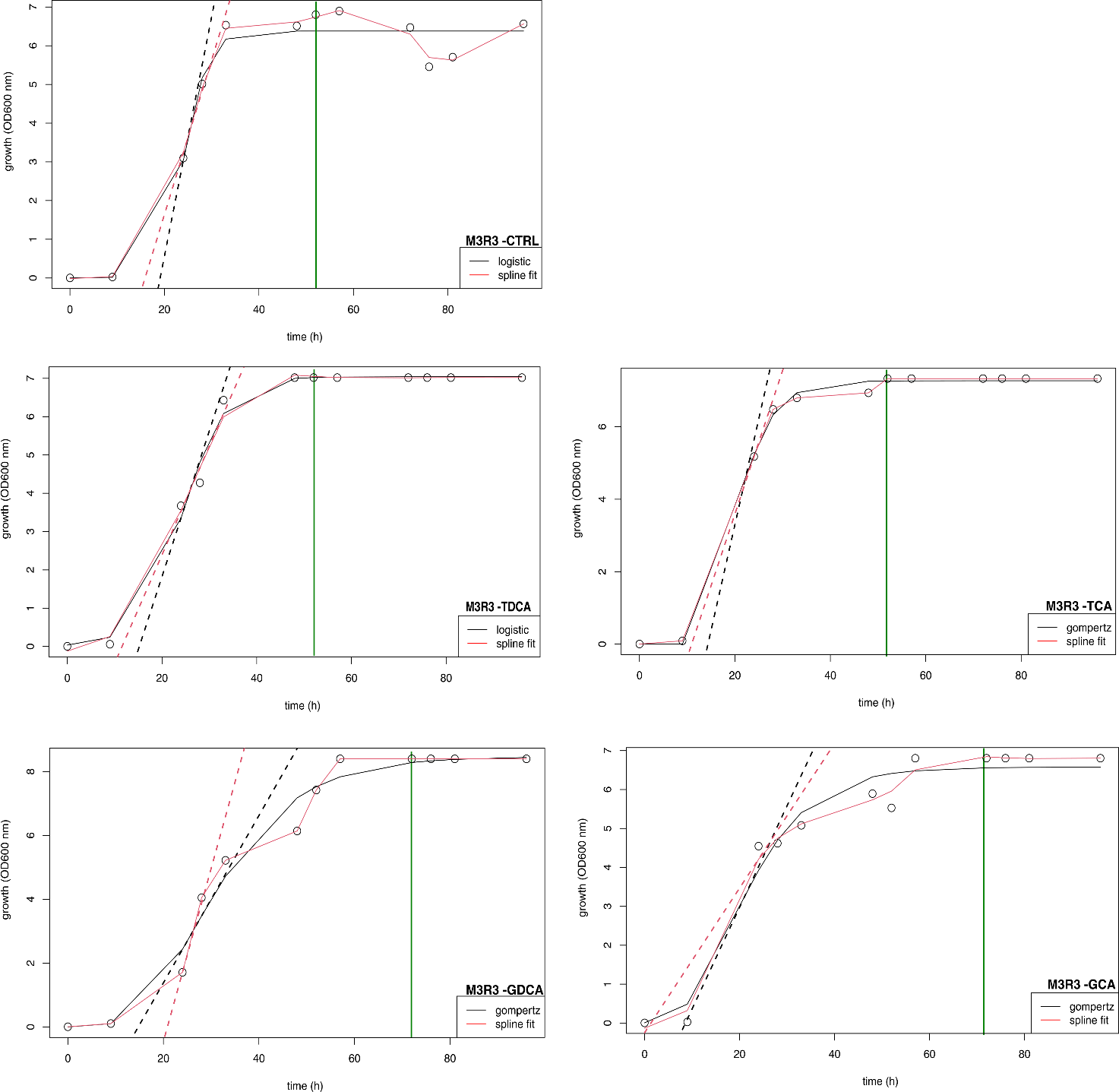


**Fig. S2** Representative growth curves of *Levilactobacillus brevis* strain M3R3 in presence of 1.0 % (*w/v*) bile salts (BSs) mixture (B) compared with the control condition (A; MRS medium). Optical density at 600 nm (OD₆₀₀) was recorded over time. Each condition was tested in three independent biological replicates. Growth curves were modeled using the Grofit package in the R environment, applying both parametric (black) and spline-based (red) methods. Black and red dotted lines indicate the intercepts corresponding to the maximum growth rate (h⁻¹) calculated by the parametric and spline-based approaches, respectively. The vertical green line marks the sampling point used for supernatant and cells collection for UHPLC/HR-MS analysis and RNA extraction, respectively.


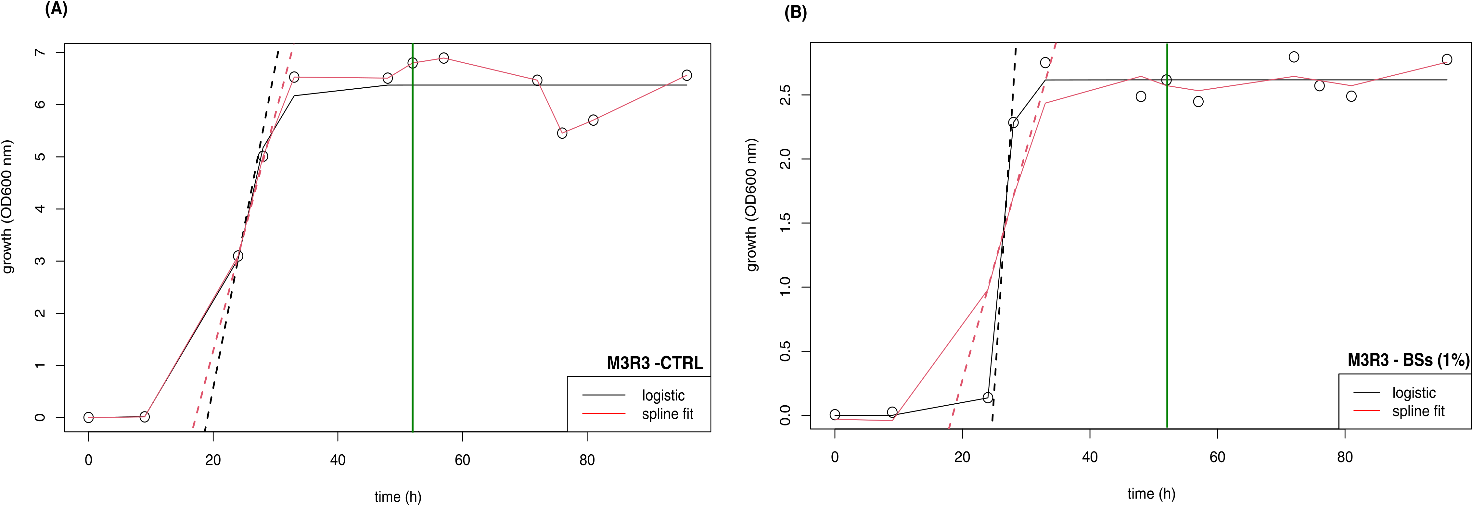


**Fig. S3** Distribution of KEGG orthology (KO) categories of identified protein-coding genes in the *Levilactobacillus brevis* M3R3 genome.


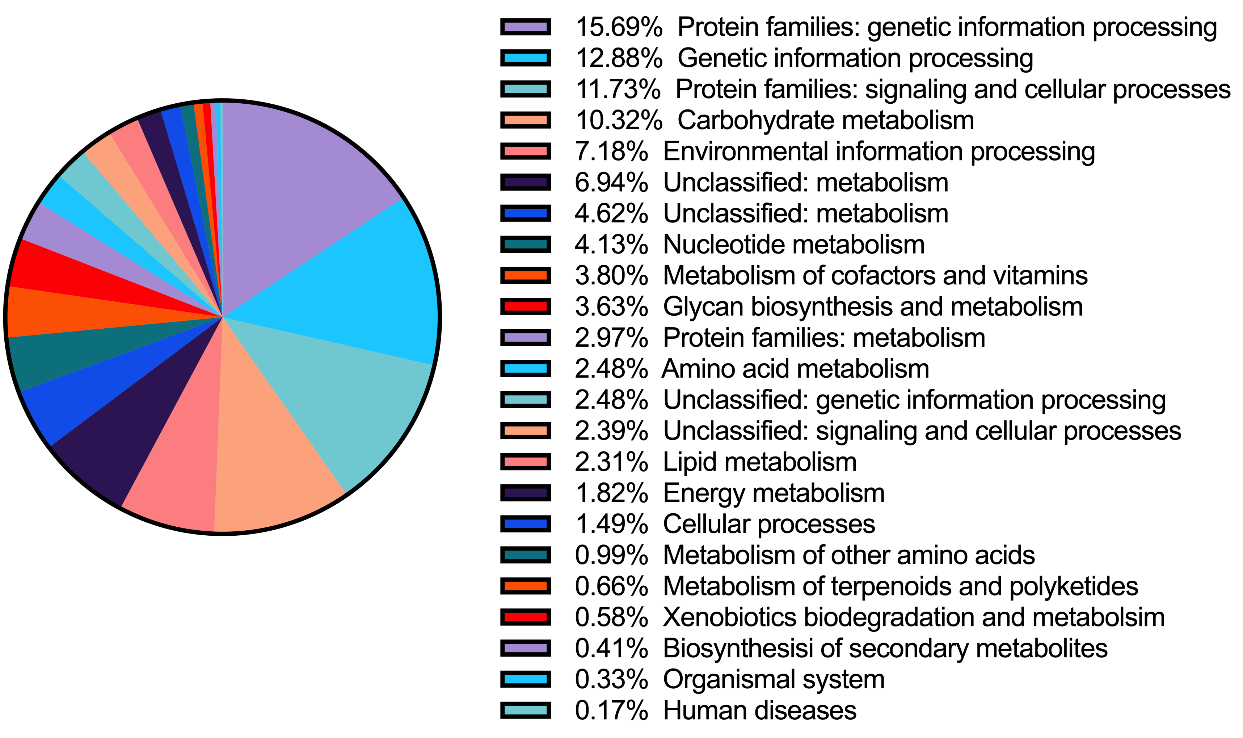


**Fig. S4** Distribution of mobile genetic elements in *Levilactobacillus brevis* M3R3 genome. Major categories considered were IE, integration/excision; RRR, replication/recombination/repair; P, phage; STD, stability/transfer/defense; and T, transfer.

**
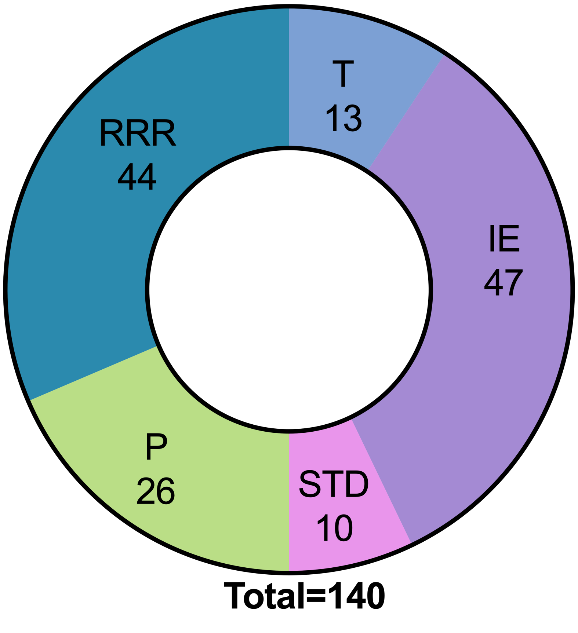
**
